# Supplementary material for: Dissecting Adaptation Mechanisms to Contrasting Solar Irradiance in the Mediterranean Shrub Cistus incanus
Source: Int J Mol Sci. 2019 Jul 23;20(14):3599. doi: 10.3390/ijms20143599 (PMC6678608; doi:10.3390/ijms20143599)
Supplement: Supplementary file 1 [file ijms-20-03599-s001.pdf]

Table S1. List of primers used for qRT-PCR of selected genes.

Transcript ID	Primer Forward	Primer Reverse	Amplicon size (bp)
DN78643	GTGCACTTGTATGGTCGGGG	GTGGGCCCGTAAAACTCACT	130
DN89715	GATCTTGCCTAGTTTGCGCG	TCCGACCTTTCTTGCTGGAC	117
DN8257	GTTCGGAACCATGAAGCAGC	AGCGTAGGAGAGTATGGGGA	115
DN95220	CCACCTCGCTGAGAACTGAG	TGGCAGCAAGAACAGACCTT	91
DN3694	ACAGGATTTGGCATCGGTGT	CGACCAACAACCCCATAGCA	127
DN100152	TTTCAAGCCATCTCCACCCC	AACAGTGGCAGCGACATGTA	120
DN91246	CCTCTTTCCGGGTCTTTGCT	CCGGGAATCTTTTGTGCACC	101
DN46243	GGTATTCCGCTCGAGTGTGT	CCTCAAAACGCCCTCCATCT	126
DN10482	AGATGTCGAAGGGTACGGGA	AGTTGATACGCGATGGAGGC	104
DN86627	GTGGGCGGTTTTCTGTGAAC	GGGTATTTCGCAGGCTCAGT	90
DN82770	GGATGACGGTGGTGATGGTT	TCGGGGCTCAAGATTTCGAC	106
DN89482	ACAAAGGGAGAGGACAGCAG	GCGAAGGAAGTGAGAACAGC	90
